# Thematic Analysis of Medical Notes Offers Preliminary Insight into Precipitants for Asian Suicide Attempters: An Exploratory Study

**DOI:** 10.3390/ijerph15040809

**Published:** 2018-04-20

**Authors:** Carol C. Choo, Roger C. Ho, André A. D. Burton

**Affiliations:** 1 College of Healthcare Sciences, James Cook University, Singapore 387380, Singapore; 2 Department of Psychological Medicine, Yong Loo Lin School of Medicine, National University of Singapore, Singapore 119007, Singapore; pcmrhcm@nus.edu.sg; 3 School of Psychology, Curtin University, Perth 6102, Australia; andre.burton@postgrad.curtin.edu.au

**Keywords:** suicide precipitants, Asia, suicide attempts, thematic analysis, stress

## Abstract

One important dynamic risk factor for suicide assessment includes suicide precipitant. This exploratory study used a qualitative paradigm to look into the themes surrounding precipitants for suicide attempts in Singapore. Medical records related to suicide attempters who were admitted to the emergency department of a large teaching hospital in Singapore over a three year period were subjected to analysis. A total of 666 cases were examined (69.2% females; 63.8% Chinese, 15% Malays, 15.8% Indians), ages ranged from 10 years old to 85 years old (Mean = 29.7, Standard Deviation = 16.1). The thematic analysis process that was applied to the textual data elicited key concepts labelled as Relationship issues, Financial strain, Socio-legal-academic—environmental stress, and Physical and mental illness and pain. Interpreted with other recent local research on suicide attempters in Singapore, the findings have implications for informing suicide interventions.

## 1. Introduction

Suicide assessment typically considers long standing chronic risk factors, in addition to the current state of the individual, to inform interventions [1,2,3,4]. In contrast to chronic risk factors such as psychiatric history, age, and gender, which are static and enduring, dynamic risk factors such as suicidal ideation are episodic and dynamic. Dynamic risk factors also include precipitant events, and intense affective states. Suicide precipitants are the problems encountered shortly before suicide, sometimes referred to as ‘triggering events’ [5]. Psychosocial disturbances can be destabilizing to vulnerable people [6] and trigger suicidal ideation or suicide attempts [7]. Many psychosocial factors occur along with psychiatric symptoms that increase suicide risk [8] such as interpersonal conflicts and legal problems [9]. In Asia, prominent psychosocial precipitants include divorce [5], unemployment [10], economic problems, and retrenchment. There is a spectrum of varying precipitants across the lifespan for Asian suicide attempters [2,3], which include interpersonal conflicts, stress with military service [11,12,13,14], family problems [10,11,15], academic stress [16], financial issues, marital problems, and physical illnesses. Cultural influences are noted for suicide attempters in the multi-ethnic Asian society in Singapore [2], with an over-representation of Indian females, and often associated with interpersonal disputes [17].

Most suicide studies were conducted on suicide deaths, and suicide notes from completed suicides [13], and recent large scale local studies on suicide attempts are quantitative [2,3]. The current study on suicide attempts is unique in that it seeks to deepen our understanding of underlying mechanisms triggering people to suicide using qualitative enquiry, to inform more targeted strategies to enhance patients’ capacity to cope with suicide precipitants. The objective of this study is to investigate the research question of what are the mechanisms underlying precipitants to suicide attempts. Thematic analysis will be conducted on assessment notes made by medical officers during the assessment interview following a medically treated suicide attempt. The themes generated could be used to deepen our insight to inform interventions. Qualitative analysis could reveal unexpected results, or unveil unique results embedded in the data.

## 2. Materials and Methods

### 2.1. Procedure

Ethics approval was obtained from the Domains-Specific Review Board of a large teaching hospital in Singapore and the Human Research Ethics Committee at James Cook University, approval number H3445. Investigations were carried out following the rules of the Declaration of Helsinki of 1975. This study is based on an archival retrospective review of de-identified hospital records of patients who were admitted for a suicide attempt from January 2004 to December 2006. Textual data was collected by examining the medical notes in multiple hospital databases related to the suicide attempters. This data set is the most comprehensive data set from the hospital, as the assessment data that addressed our research question was not available prior to and following the stipulated period.

All cases of attempted suicide were assessed by medical officers in the emergency department under the supervision of a consultant psychiatrist, and the interview took approximately 20 min. This assessment was part of the protocol standard operating procedure for patients admitted following a medically treated suicide attempt. The inclusion criterion for the current study were patients who were admitted to the emergency department from January 2004 to December 2006. There were a total of 666 cases examined in the study. Textual data was extracted from multiple databases. Available information about the suicide attempt, as well as information about the suicide attempters, and suicide precipitants were extracted by the lead researcher, who is an experienced mental health clinician.

### 2.2. Approach

In order to address the gaps in literature, it was decided this study would focus on identifying themes from notes recorded by medical doctors during the psychosocial assessment, and related data would also be collected from multiple hospital databases. This would provide the researchers with scope for further investigation of the subject in question, and the most appropriate method of analysis would be a thematic analysis. Thematic analysis offers flexibility and theoretical freedom, and is comparable with the constructionist paradigm in examination of the range of experiences within the contemporary society [18]. However, there have been criticisms of this approach due to the lack of clear guidelines for researchers employing such methods [19]. The researchers in this study decided to employ a clear, replicable, and transparent methodology, as detailed by Braun and Clarke [19]. To the best of our knowledge, this exploratory study using a qualitative paradigm has not been conducted in the local Asian context. An inductive ‘bottom-up’ approach would be taken, where the themes identified would be strongly linked to the data [19].

### 2.3. Cases

666 patients were admitted for a suicide attempt from January 2004 to December 2006. (69.2% females; 63.8% Chinese, 15% Malays, 15.8% Indians), ages ranged from 10 years old to 85 years old (Mean = 29.7, Standard Deviation = 16.1). In this sample, 6% had a formal psychiatric/medical diagnosis during the time of evaluation. Of those, 41% were diagnosed with depression, 18% were diagnosed with substance abuse, 10% were diagnosed with adjustment disorder, 8% were diagnosed with schizophrenia and borderline personality disorder respectively, 5% were diagnosed with chronic medical illness, and 3% each were diagnosed with acute stress reaction, bipolar disorder, post-traumatic stress disorder, and alcohol abuse, respectively. The majority of the patients (78.5%) overdosed in the suicide attempt.

### 2.4. Data Analysis

Six phases of analysis were employed [19]: familiarizing with the data, generating of initial codes, searching for themes, reviewing themes, defining and naming themes, and final reporting using selected extracts. The textual data available in multiple databases relating to the 666 suicide attempters was re-read several times [19], resulting in data immersion, before progressing into the coding phase. The coding involved an inductively driven process, whereby the lead researcher read and re-read the textual data. Codes identified the most basic features of the raw data, such as key words and phrases that appeared relevant and pertinent to the research question of suicide precipitants. The coding process involved a constant moving backward and forward within the textual data set, in order to analyze the extracts that had been initially identified. Rigorous note taking was undertaken by the lead researcher in the coding process, and coding schemes were identified through the annotation of ideas. The codes were then sorted according to their similarity, via a thematic map, into identified themes, which would serve as the units of analysis. Later reviews were made to ensure no codes had been omitted.

All initial codes relevant to the research question were incorporated into a theme. A theme captures something important and meaningful within the data set in relation to the overall research question [19]. In the context of this study, a theme had to relate to our research topic on precipitants to suicide attempts.

Once a set of candidate themes had been established, the refinement of the themes was necessary. Consistent with expert guidelines [19] and previous relevant research [20], this process involved reviewing the collated codes and extracts, looking for internal homogeneity, and considering whether the candidate themes formed a coherent pattern and accurately reflected what was evident in the data set as a whole. Redundant themes were discarded, such as those that did not fit the research question or did not appear to have coherence of meaning. Moreover, identifiable relationships, links, and distinctions between themes were detected. The practical basis for this thematic refinement process was to ensure that the themes were all broadly related to one another, yet did not overlap too closely in their content. Conceptually, this meant that the themes were able to stand in their own discernable categories with regards to the original patterns that had emerged in each. The next stage involved defining and naming the themes, with clear definition accompanied by a detailed analysis. The final stage involved choosing examples to illustrate elements of the themes. The extracts will be included in the next section, these extracts were selected as they clearly identified issues within the theme and presented a lucid example of the point being made.

## 3. Results

The thematic analysis process that was applied to the textual data elicited key concepts that were evident in the data. The codes were categorized into the following four themes. The subsections below include the extracts from the medical notes which captured the essence of the respective theme without unnecessary complexity [19].

1. Relationship issues
2. Financial strain
3. Socio-legal-academic—environmental stress
4. Physical and mental illness and pain

### 3.1. Relationship Issues

This theme is defined by interpersonal conflicts and arguments with colleagues, classmates, and family. Having just broken up or in the process of breaking up from an intimate relationship or marriage were common precipitants among the suicide attempters. In addition, the ‘suspicion’ and ‘fear’ that the spouse was having an extramarital affair was mentioned by 5 of the suicide attempters. Extracts from direct quotes were appended below to illustrate this theme.

Amy: scolded by mother

Beryl: quarrelling with children, husband past two weeks

A.L.: unhappy with parents quarrelling

Denise: conflict with colleagues, conflict with boyfriend

T.L.: weekly arguments with classmates

Carly: quarrel with husband; threatened divorce

### 3.2. Financial Strain

The theme is a conglomerate of stress stemming from ‘unpaid bills’, ‘debts’, ‘bankruptcy’, ‘credit card loans’. A striking feature about this theme was that ‘unemployment’ was commonly mentioned. Analysis revealed other reasons for financial-related stress, including ‘gambling debts’, ‘retrenchment’, and ‘business failure’. In addition, lack of family support seemed to exacerbate the problem of financial strain.

L.T.: lost in gambling, tried to borrow from mother but failed

### 3.3. Socio-Legal-Academic and Environmental Stress

The theme is a conglomerate of problems stemming from academic stress, legal problems, problems with mandatory military service, and other concerns arising from the psychosocial environment. Two suicide attempters mentioned ‘recent sex change’ and ‘homosexual tendencies’, and one of them worried about having to return to military camp. Moreover, childhood sexual abuse, sexual and physical assault by a close friend or family member were mentioned as well as an inability to cope. Several suicide attempters mentioned about stress associated with grief from a family member who died recently, and the anticipated grief related to the spouse recently being diagnosed with a terminal illness.

### 3.4. Physical and Mental Illness and Pain

The theme is a conglomerate of subjective distress stemming from chronic physical and mental illness, history of anxiety and depression, sleep problems, substance abuse, and legal problems.

G.L.: intractable pain

K.T.: felt depressed; insomnia

P.T.: headache for one month

## 4. Discussion

The exploratory study examined underlying themes for suicide precipitants in Singapore using a qualitative paradigm. Medical records related to 666 suicide attempters were analyzed. As hypothesized, themes emerged to address the research question of mechanisms that underlie suicide precipitants. The results detailed above highlighted important findings regarding the complex interplay of precipitants that drove people to suicide. The textual data extracted from the medical records reflected a myriad of issues such as history of mental and physical illness, interpersonal conflicts, as well as legal, academic, financial and work problems, coupled with the inability to cope seemed to intersect and precipitate suicide attempts. Both the chronicity of the health problems as well as the acuteness of recent psychosocial stressors surfaced. The findings are consistent with previous research relating suicide with precipitants such as medical problems [21], work/academic problems [16], family problems [22], relationship problems [23], and financial problems [24]. The findings are also consistent with previous research associating suicide attempts with lack of effective coping strategies to manage life stressors [25], and chronic stress [26]. People who are more vulnerable to attempt suicide are also more vulnerable to become more distressed by stressful life events because they lack certain types of coping strategies (direct action, situation redefinition, and acceptance) with a poorer outcome in stress reduction.

The study also unveiled the stresses faced by sexual minorities in the Asian society. This finding is congruent with the increased recognition that in recent times, that psychosocial stresses for suicide attempters include homosexual orientation, and identification with sexual minority or ‘non-dominant’ culture [27].

The perception of ‘pain’ mentioned by the suicide attempters could be interpreted as physical pain, psychosomatic pain, or the subjective distress and psychological pain which might present during a crisis triggered by stressful life events [28]. During such stressful periods, negative emotions are heightened, and psychological anguish follows. Vulnerable people could be affected by perturbation or subjective distress caused by physical or psychological pain [28], and a belief that there is no other option besides suicide [29], as suicide puts an end to thwarted psychological needs and intolerable emotions [28]. The suicide attempters focused on negative emotions and stressors, which seemed to suggest a reduced capacity to engage in problem solving, stress management [30], or emotional regulation [1]. Therapeutic interventions from a clinician could involve the establishment of an empathic bridge into the suicidal patient’s subjective distress by reflecting the feelings of acute depression, grief, and other powerful emotions. This could help the suicidal patient hold the pain long enough to understand and process it, rather than culminate in suicide attempt to eliminate the psychological distress [31]. By reducing the anguish and perturbation, lethality of suicide could be reduced [28]. By forming a mutual understanding of shared meaning of the underlying mechanisms affecting perturbation from suicide precipitants, the clinician could help vulnerable patients explore more effective coping or problem-solving skills [10], so they could feel more confident and in control of their problematic situations.

Limitations of the study included the reliance on self-report, the brief nature of the assessment, manual analysis of the data, as well as the dated data set. The clinical assessment took place in a busy emergency department setting, the interviews were brief and medical notes were succinct, and might not fully capture the richness of the suicide attempters’ narratives. More in-depth interviewing in future research could strengthen the comprehensiveness of the data. Future research could seek consent from patients to interview family members or spouses, with more in-depth interviewing to elicit deeper understanding, with software such as NVivo version 10 (QSR International Pty Ltd., 2014) to help with qualitative analysis of the data, and collection of more recent data. The preliminary findings on sexual minorities suggest a need for further research into this group. Such research might enhance our understanding of the lived experience as a sexual minority in the Asian society and give an indication of whether there are current gaps in service delivery.

Our exploratory study offers preliminary evidence and insight into suicide precipitants for Asian suicide attempters in Singapore. Interpreted with other recent local research on suicide attempters in Singapore [1,2,3], the findings have implications for informing suicide interventions. To enhance the therapeutic effectiveness of suicide interventions, the findings suggest that clinicians could build an empathic bridge into the suicidal patient’s subjective distress by accurately reflecting their feelings of acute depression, grief, and other powerful emotions [1]. Clinicians could assist patients to cope and engage in emotional regulation [1] by using techniques informed by Dialectical Behavioural Therapy to manage stresses related to relationship issues, financial strain, environmental stress, and physical and psychological pain. This study adds to the current suicide literature, by deepening our insight into mechanisms underlying suicide precipitants. It also highlights the stresses affecting sexual minorities in Singapore, and draws further attention to the necessity of training competent and empathic clinicians to work with these vulnerable patients.

## 5. Conclusions

The thematic analysis process that was applied to the textual data elicited key concepts labelled as Relationship issues, Financial strain, Socio-legal-academic—environmental stress, and Physical and mental illness and pain. Interpreted with other recent local research on suicide attempters in Singapore [1,2,3], the findings have implications for informing suicide interventions.
